# Music recharges people: Synchronized music during aerobic exercise leads to better self-regulation performance

**DOI:** 10.1371/journal.pone.0278062

**Published:** 2022-12-21

**Authors:** Chenyang Li, Chengji Jin, Ziyun Zhang, Peng Shi

**Affiliations:** 1 Physical Education Institute, Liaoning Normal University, Dalian, Liaoning Province, People’s Republic of China; 2 School of Physical Education and Sports Training, Shanghai University of Sports, Shanghai, People’s Republic of China; Sport Sciences School of Rio Maior - Politechnic Institute of Santarem, PORTUGAL

## Abstract

Previous studies have demonstrated that music has a positive effect on individuals during exercise and sports. We speculate that one of the mechanisms for this positive effect may be that music reduces the consumption of self-regulation strength. The primary objective of this study was to use a self-regulation strength model to explain the impact of music on individuals during aerobic exercises. Specifically, we examined the effects of synchronous music on college students’ depletion of self-regulation during aerobic exercises. The participants underwent a pre-test in which they had to maintain 50% maximum voluntary contraction (MVC) isometric grip and do exercise planning tasks. For subsequent power bicycle riding (aerobic exercise), the participants were divided into a music group and a control group. The music group performed aerobic exercises with synchronous music, while the control group performed aerobic exercises without music. After aerobic exercise, the participants underwent a post-test for isometric grip and exercise planning tasks. The results showed that the music group planned to reduce their efforts less for an upcoming exercise period (*p* < 0.01, *d* = 0.81), and their wrist flexor muscle group generated less electromyographic activation during an isometric grip task that maintained 50% MVC (*p* < 0.05, *d* = 0.80) than the control group. However, the two groups showed no difference in the duration of 50% MVC. This shows that: (a) for the same duration, participants in the music group required a lower degree of muscle activation than the control group, suggesting that music reduced the consumption of self-regulation strength in aerobic exercise; and (b) music decreased participants’ planned exertion declined, also suggesting that music reduced the consumption of self-regulation strength in aerobic exercise.

## Introduction

A large number of studies have investigated the positive effects of music on individuals in exercise and sports [1–6]. First, regarding physiology, music can increase the neuromuscular fatigue threshold of the quadriceps femoris muscles in incremental single-leg knee extensors [7]. Further, blood lactate concentrations and oxygen consumption were lower with music for submaximal running [8, 9]; Second, regarding psychophysics, music can reduce overweight and obese women’s perceived exertion during self-selected space walking [10]. Third, regarding physical performance, music can increase total work, relative peak power output, peak power output and mean power output in the Wingate Anaerobic Test [11, 12]. Also, time-to-exhaustion is longer when running while listening to music [9]. Fourth, regarding psychology, music can promote a positive affect during aerobic exercise [13–15]. However, the underlying mechanisms remain to be systematically explored. Current studies on the mechanism include the following. First, the capacity of afferent neurons is limited, and there is a dynamic and continuous competition between the body’s external and internal information for brain processing. Focusing on external surroundings (music) can inhibit an individual’s perception of physiological feedback signals related to physical exertion and, to some extent, shield against the perception of fatigue [16]. When beginning to exercise, individuals tend to focus on the external environment. With the accumulation of fatigue, physiological signals become stronger, and individuals’ focus turns from focusing on the external to focusing on the internal (physiological feedback signals). Karageorghis and Jones [17] found that the switching point of individuals’ attention from the external to internal was delayed by 10% of the maximal heart rate reserve in a music condition. Second, the central pattern generator in the brain may be used to regulate time function and control rhythm response; human beings naturally tend to synchronize movement with rhythmic music [18]. Musical rhythm can stimulate the central pattern generator to produce a stable rhythm pattern, thereby reducing the participation and feedback of the higher cortex, which has a lower oxygen consumption rate [19]. No systematic psychological theory explains the mechanism of the positive effect of synchronized music on aerobic exercise. We attempted to understand the mechanism of the positive effect of music on individuals during exercise, using a self-regulation strength model.

According to Baumeister et al. [20, 21], self-regulation can be described as a process in which an individual tries to consciously control or overthrow the dominant behavior or reaction tendency to achieve a specific aim. Baumeister et al. proposed a self-regulation strength model to explain human self-regulation behavior [22, 23]. First, the model conceptualizes self-regulation as a global but limited resource. All self-regulation behaviors utilize the same finite amount of self-regulation strength [24–26]. Self-regulation strength is limited, and is discharged when people try to control their behaviors, thoughts, or emotions [21, 27]. Second, after completing the self-regulation task, strength is temporarily exhausted without immediate replenishment, which is called self-regulation depletion [28]. Self-regulation depletion leads to self-regulation failure. Third, self-regulation strength depletion is reduced through rest and frequent self-regulation [29]. Self-regulation abilities are important determinants of exercise performance, such as predicting and making plans to overcome exercise obstacles [30], developing exercise plans and calendars [28, 31], and handling aches and discomfort in physical activity [32]. The depletion of self-regulation strength leads to a decline in physical activity performance. If music can reduce the consumption of self-control strength, it can improve physical activity performance.

A previous study showed that music accompanying aerobic exercise can promote a positive affect [33] and that a positive affect can reduce the depletion of one’s self-regulation strength [24, 34]. This study posits that the positive affect that is caused by music during exercise can reduce the consumption of self-regulation strength, which may be one of the reasons music improves exercise performance. This may apply not only to aerobic exercise but also to high-intensity interval training (HIIT) and resistance exercises. In terms of different exercise modes, HIIT in particular, Stork et al. [35] found that participants who listened to music had exercise enjoyment that increased with time, and was always higher than that without music. This indicates that the participants were happier performing HIIT with music. Therefore, the positive affect of music reduces the depletion of self-regulation strength and may also apply to HIIT. Although no research has directly investigated the impact of music on the affect of resistance exercise, some studies have shown that the music group has a lower rating of perceived exertion (RPE) during persistent exercise [36]. The positive valence of individual affect increase with a decreases in RPE during exercise [37]. This may indicate that the smaller the RPE value of the music group in the resistance exercise, the more positive their affects. Self-regulation depletion reduces one’s endurance performance and power output [38–40]. During resistance exercise, exercising without music showed lower strength endurance (repetitions to failure at 60% 1-RM) [41] and maximal power output (incremental single-leg knee extensor.) [7], similar to the performance of self-regulation depletion. This could mean that the no-music group consumed more self-regulation strength than the music group during the resistance exercise. Therefore, if this study proves that music has an impact on self-regulation strength, the results can be applied not only to aerobic exercise, but also to HIIT and resistance exercise.

Our primary purpose was to discuss the impact of synchronous music on college students’ self-regulation depletion during aerobic exercise using an isometric grip and exercise planning task, which are classic tasks for measuring self-regulation [42, 43]. Research on the impact of music on individuals during exercise divides music conditions into synchronous music conditions in which the music rhythm was synchronized with movement speed, and asynchronous music conditions in which the music rhythm was not synchronized with movement speed. In these studies, synchronous music had a greater positive impact on individuals [19, 44, 45], so it was selected for this study. According to the self-regulation strength model, depletion of limited self-regulation strength can reduce exercise performance [23, 46, 47]. Music can improve exercise performance by reducing the consumption of self-regulation strength. Therefore, we assumed that the music and control conditions in the power bicycle task caused different levels of self-regulation depletion. Specifically, compared to the control group, the music group with lower levels of self-regulation depletion shows less performance decline in the isometric grip task and planned to reduce less effort in the exercise plan.

## Materials and methods

### Research design

Our empirical study used a single-factor between-subject experimental design [48].

### Participants

The participants were recruited by research assistants who attended college courses and invited students to participate. Participants received 100 RMB (USD $14.95) within three days of the end of the experiment, and a small gift immediately after the end of the experiment in appreciation of their participation. Participants were retained if they met the following five inclusion criteria: (a) no orthopedic, cardiovascular, or respiratory disease restrictions that could hinder exercise; (b) no anabolic steroids or other drugs that could improve athletic performance; (c) no regular cycling training; (d) no recent isometric grip participation and (e) haven’t heard the music material “I Fly in My Dreams” before. We were concerned that the two groups of participants would be homogeneous in terms of physical activity and aerobic fitness. The amount of physical activity was measured using the Physical Activity Rating Scale 3 (PARS-3). The PARS-3 examines the amount of physical activity from three aspects: intensity, time, and frequency of physical activity. The scale uses three items focusing on physical activity intensity, duration of physical activity, and frequency of physical activity. For each item, five choices were listed, with points from 1 to 5 for quantification. The calculation of the physical activity score is based on the following equation: the amount of physical activity = intensity (1–5) × time (0–4) × frequency (1–5), and the score ranged from 0 to 100. The test–retest reliability of the PARS-3 was 0.82 [49]. Aerobic fitness was negatively correlated with increased BMI [50]. We measured participant’ BMI to assess aerobic fitness, BMI using the following formula: BMI = weight (kg) ÷ height (m)^2^.

Based on the effects observed in previous studies have examined the impacts of self-regulation depletion during performance in exercise tasks [42, 43]. We expected Cohen’s *d* = 0.65 (medium size-effects: 0.5 < *d* < 0.8). To achieve 80% power to detect significant differences between the groups, 30 participants in each condition were required, with α = 0.05. The randomization sequence was created using a computerized random number generator in MS Excel with 1:1 allocation, and participants were categorized into two groups. The study sample consisted of 61 healthy participants (music group = 31, 16 of whom were women; control group = 30, with 15 women). The demographic characteristics of the two groups were similar, and no significant differences were reported in age, *t* (59) = 1.06, *p* = 0.29, *d* = 0.26; BMI, *t* (59) = 0.41, *p* = 0.68, *d* = 0.15; or the amount of physical activity, *t* (59) = -1.42, *p* = 0.16, *d* = 0.37 (Table 1).

**Table 1:** Means and standard deviations for demographic characteristics. *Mean* (*SD*).

	music(*n* = 31)	control(*n* = 30)
Age (years)	21.1 (1.2)	20.8 (1.0)
BMI (kg/m^2^)	22.6 (1.8)	22.4 (1.9)
The amount of physical activity (score)	45.3 (8.1)	48.6 (9.9)

Informed consent and research were approved by the Institutional Review Board of Liaoning Normal University (registration no. LL2021-065) and was conducted in accordance with the Declaration of Helsinki [51–53], which sets out the basic ethical principles of human research. All participants signed written informed consent forms before the experiment and were told not to do any form of strenuous exercise within 24 h before the test, sleep for at least 7 h, and not to eat or drink (except water) within 2 h of the test.

## Methods and measures

### Isometric grip task

The reduction in self-regulation strength decreased the performance of the isometric grip task, which was used to measure self-regulation in actual physical activity [34, 43]. The change in isometric grip contraction time throughout the two endurance tasks test was the first dependent variable, during which the participants maintained a 50% MVC. The participants performed the isometric grip task both before and after the power bicycle task, as in the pre- and post-tests. Based on the procedure used by Bray et al. [43], participants used an isometric grip dynamometer (model MLT003/D; AD Instruments) with a graphic computer interface (PowerLab 4/25T; AD Instruments) to perform an endurance isometric contraction of their preferred hand at 50% of their MVC. The participants performed the MVC twice for 5 s on the dynamometer before each endurance trial, and determined the standard of 50% MVC based on the average of the maximum value of the two MVCs. Participants squeezed the handgrip dynamometer and received feedback in the form of a force tracking on a computer monitor (a real-time graphed line indicating how much force was being generated) when performing endurance contractions. The target force (50% MVC) was displayed on the screen as a static line. Participants were asked to squeeze their hands for as long as possible to keep the force tracking line at, or above, the target level. The researcher reminded the participants that the test ends when the grip strength is lower than 50% MVC for more than 1 s. The physical performance-dependent variable was the number of seconds required for the participants to maintain isometric grip strength at ≥ 50% MVC.

### EMG and force recording

The change in the surface electromyographic (EMG) activation across the two trials of the isometric grip task was the second dependent variable. During the isometric grip task, we successively monitored the strength output of the hand and EMG activity of the wrist flexor muscle group [43]. First, an alcohol swab was used to clean and abrade the front surface of the forearms. A disposable 3M recording electrode (diameter, 1 cm) was fixed above the abdomen of the forearm flexor group. We placed the reference electrode in the tendon region on the front surface of the forearm, approximately 4 cm from the stigmatic electrode, and placed the ground electrode on the epicondyle of the internal humerus. The Powerlab 4/25t data acquisition system (AD Instruments) amplified, digitized, and continuously streamed EMG signals to a PC at a sampling rate of 4 kHz and bandpass filtered at 10 Hz to 1k Hz. Integrated EMG (iEMG) analysis was conducted to quantify of muscle activity using Chart 7 software (AD Instruments Pty. Ltd., USA). To standardize the iEMG generated by each participant during the endurance trials, we calculated their average iEMG in five 1-second windows at the start of the trial (baseline) and at each quartile (25%, 50%, 75% and 100%) of the total time to failure, to show the amount of iEMG generated throughout each trial [43]. During MVC measurement, a 1-s peak EMG (mV) was selected as a normalizing value (100%). To calculate% MVC, iEMG (mV/s) was divided by the peak amplitude of the MVC (mV/s) for standardization [54–56].

### Exercise planning task

The reduction in self-regulation strength decreased the exertion of their planned exercise, and an exercise planning task was used to measure self-regulation in planned physical activity [42]. The third dependent variable was the changes in participants who planned to exercise for 30 min in two trials of the exercise planning task. The participants completed two exercise planning task measurements before and after the power bicycle task as the pre- and post-tests. Based on Martin Ginis and Bray [42], in the pre-test, participants were told that they would try new exercise training equipment later in the experimental phase. They were asked to develop a 30-minute exercise circuit, which included six five-minute bouts of exercise, and they chose one piece of equipment every five minutes. The researcher provided participants with a list. The letters “A” to “J” on the list referred to 10 pieces of sports equipment. The required intensity for each piece of equipment was marked with RPE values of 1, 2, 3, 4, 5, 6, 7, 8, 9, and 10. RPE values were obtained using Borg’s CR-10 RPE scale [57], which is a numerical method for measuring perceived exertion. The higher the RPE value, the greater the effort required to use the equipment. Then, a six-space exercise plan list for six five-minute exercises was provided to the participants, and they were asked to choose a piece of equipment during each five-minute exercise and write the corresponding letter in each space. Participants could repeat the choice of equipment as needed. For example, a participant may plan to select C, D, E, F, E, and D in cycles, which translates into RPEs of 3, 4, 5, 6, 5, and 4, respectively, for each exercise. According to [(3 + 4 + 5 + 6 + 5 + 4)/6], the planned exertion in the 30-minute circuit training is 4.5. This measures the baseline level of participants who planned to invest in physical exertion during a 30-minute exercise period. Later, in the post-test, we informed participants that half of the participants used sports equipment “A” to “J,” but they were randomly assigned to use sports equipment “K” to “T.” These are different pieces of equipment, but each piece corresponds to the PRE value (as with pieces “A” to “J”). The participants were required to create a circuit training plan. Changing the letters of the equipment shielded the interference of memory and restrained the residual bias from the pre- to post-test. The participants were unaware that they would not actually perform the exercises.

### Aerobic exercise program

The standard intensity of aerobic exercise was determined according to existing literature and the physical characteristics of the college students. Since the difference in individual basic heart rate is not considered by the maximum heart rate (HR_MAX_) [58], we used the reserve heart rate to confirm the exercise intensity. The reserve heart rate is equal to the maximum heart rate minus the resting heart rate, where the maximum heart rate is 207–0.7 × age [59]. Participants were seated in individual cubicles and were instructed to sit quietly and relax during resting heart rate assessment. resting heart rate was assessed at rest for 10 minutes using a Polar Pro Trainer 5.0. The target heart rate was calculated as follows: target heart rate = resting heart rate + reserve heart rate × intensity%. Based on the continuous training method in sports training, moderate-time continuous training is 10–30 min, the heart rate is maintained at approximately 160, and the individual’s energy supply stems primarily from aerobic metabolism energy supply [60]. Therefore, according to moderate-time continuous training, the intensity of the target heart rate calculated in the aerobic exercise task of this study was set to 75%, and the participants’ heart rate reached approximately 160 beats per minute (BPM). Power bikes (Custo med, ec3000e) were used to implement aerobic exercise programs, and a heart rate strap (Polar Pro Trainer 5.0) was used to monitor participants’ heart rate during exercise. The resistance of the power bicycle was adjusted according to the target heart rate; the resistance range was 30–200 W, and the exercise rhythm was 75 revolutions per minute (RPM) [45]. There was a five-minute warm-up in the aerobic exercise task; the intensity of exercise should reach the target heart rate in these five minutes and maintain it there while exercising for 20 minutes; followed by a cool-down for five minutes. The heart rate of the participants gradually became slower than the target heart rate, and the exercise speed slowly approached 0 RPM during the cool-down period. The two groups reported no significant differences in maximal heart rate, *t* (59) = -1.06, *p* = 0.29, *d* = 0.28; resting heart rate, *t* (59) = 1.16, *p* = 0.25, *d* = 0.30; and target heart rate, *t* (59) = 0.96, *p* = 0.34, *d* = 0.25 (Table 2). This suggests that the intensity of aerobic exercise did not differ between the groups.

**Table 2:** Means and standard deviations for heart rate. *Mean* (*SD*).

	music(*n* = 31)	control(*n* = 30)
maximal heart rate (bpm)	192 (0.8)	192 (0.7)
resting heart rate (bpm)	73 (10)	70 (9)
target heart rate (bpm)	162 (2)	162 (2)

### Music material

Referring to Lim et al. [45], “I Fly in My Dreams” was selected as the music material because of its prominent beat and relative unfamiliarity among Chinese audiences. Therefore, the influence of social and cultural background, age, music preference, and associations outside of music can be controlled. The audio editing software Logic adjusted the track speed from 156 to 150 BPM, and the sound intensity was standardized and maintained at 80 dB.

### Manipulation checks

To show that the depletion of self-regulation (and not any other variables) is driving the effects, we applied a manipulation check to examine subjective self-regulation depletion [61, 62]. The manipulation check is considered by some as informative regarding internal and construct validity [63, 64].

Referring to the manipulation check of self-regulation in a previous study [65], a seven-point Likert-type scale was used to evaluate the effort, difficulty, and comfort of the participants after performing the aerobic exercise program, to explore whether music effectively induced less self-regulation depletion during exercise. The specific questions were as follows: (a) “How much effort do you think it will take to accomplish this task?” (1 = small effort to 7 = very large effort); (b) “What do you think of the difficulty of completing the whole task?” (1 = very easy to 7 = very difficult), and (c) “How uncomfortable are you to complete the task?” (1 = very comfortable; 7 = very uncomfortable). Manipulation check was measured only once after exercise.

### Procedures

The participants were told that they would use new aerobic training equipment in the laboratory and complete a power bicycle ride. At the end of the experiment, the participants were told that the true purpose of the experiment was to explore the influence of music on exercise. It was explained that telling them the true purpose of the experiment would have affected the internal validity of the experiment as they may have imply that music would have negative or positive effects in the experiment.

After signing the informed consent form, the participants wore a polar meter to collect heart rate data, and wore electrode patches to collect EMG data. The participants then completed a pre-test of exercise planning and isometric grip tasks. Next, the participants were divided into music and control groups, following the principle of random allocation.

Music and control groups were tested in the same experimental environment. The air conditioner maintained an indoor temperature of 21°C, illumination of 300–500 lx, and noise of no more than 40 dB. Before the experiment, the researchers explained the exercise requirements of the warm-up, formal experiment, and cool-down period to the participants. To explore the effect of music on self-regulation, the music group exercised accompanied by music during the warm-up and the formal experiment. The control group exercised without music or in quiet conditions. Both groups experienced three stages: five minutes of warm-up, 20 minutes of the formal experiment, and five minutes of cool-down.

Before the power cycling task, the participants adjusted their seat height and practiced a warm-up activity. During the five-minute warm-up period, the power bicycle load was controlled by the researcher to gradually increase the heart rate of the two groups to the target heart rate. There was no music in the first two minutes of the warm-up in the music group; the participants were familiar with and adapted to power cycling. The music track “I Fly in My Dreams” was played during the last three minutes of the warm-up. The participants gradually adapted to movement patterns in which the exercise speed (75 RPM) was synchronized with the music speed (150 BPM), and each beat of the music matched the half-cycle pedal in the last three minutes of the warm-up. After the warm-up, the music group was accompanied by the same music track at a speed of 150 BPM, and the power cycling task was maintained at 75 RPM for 20 min in the formal experiment. The music group maintained pedal frequency through the rhythm of the auditory stimulation. The control group was familiar with and adapted to power cycling during the first two minutes of the warm-up. In the last three minutes of the warm-up, the control group adapted to a speed of 75 RPM according to the digital display. After the warm-up, the control group maintained an unchanging speed of 75 RPM for 20 minutes during the formal experiment. The control group was not accompanied by music, and was maintained at a constant speed using a digital display. The speeds of the two groups were continuously recorded and monitored to ensure that any deviation from the specified speed (± 2 RPM) could be corrected immediately. During the 20 minutes of the formal experiment, the heart rates of the two groups remained near the target heart rate (± 5 BPM).

The speed of the power bicycle gradually decreased to 0 during the five-minute cool-down period after the formal experiment. The participants dismounted the bicycle, conducted manipulation checks, and completed a post-test for exercise planning and isometric grip tasks.

### Statistical analysis

The study used a randomized-conditions between-subjects experimental design. Referring to the statistical analysis of a previous study, we compared the difference in self-regulation between different levels of independent variables, comparing the change between the pre- and post-test using a two-tailed independent sample t-test [42]. The change in duration (ΔDuration) was calculated by subtracting the post-test duration from the pre-test duration. The change in EMG (ΔEMG) was obtained by subtracting the post-test EMG from the pre-test EMG. The change in the exercise plan (ΔRPE) was obtained by subtracting the post-test exercise plan from the pre-test exercise plan. The independent variable was the group, and the dependent variables were ΔDuration, ΔEMG, ΔRPE, and manipulation checks (mental effort, difficulty, and comfort). The data homogeneity test using Levenes’ Test. Effect sizes were calculated using Cohen’s *d*, with *d* < 0.2, 0.2 < *d* < 0.5, 0.5 < *d* < 0.8, and 0.8 < *d* representing small, moderate, medium, and large effect sizes [66, 67].

The Kolmogorov–Smirnov test showed the following data distribution: for ΔDuration, *p* = 0.019, skewness = 0.38, kurtosis = -0.64; for ΔEMG, *p* = 0.052, skewness = -0.35, kurtosis = -0.94; for ΔRPE, *p* = 0.002, skewness = 1.13, kurtosis = 2.14; for mental effort, *p* = 0.00, skewness = 0.03, kurtosis = -0.74; for difficulty, *p* = 0.00, skewness = 0.32, kurtosis = 0.60; and for comfort, *p* = 0.00, skewness = -0.68, and kurtosis = 2.45. According to Mishra et al. [68], a distribution is called an approximate normal if the skewness or kurtosis of the data is between −1and +1. Therefore, the ΔEMG, ΔDuration, mental effort, and difficulty data follow a normal and approximate normal distribution. Because the distributions of the ΔRPE data deviated from a normal distribution, these data were logarithmically transformed using a log10 scale prior to statistical analysis. The log-transformed variables were determined to follow an approximately normal distribution (skewness = -0.13, kurtosis = -0.97). The data that followed a normal and approximate normal distribution were evaluated using an independent-samples t-test to test the effects of the group (music, control). The data for the comfortable deviation from a normal distribution were evaluated using the Mann–Whitney U test. The analyses were conducted using IBM SPSS Statistics version 23 (IBM SPSS Inc., Chicago, IL, USA). The level of significance was set at *p* < 0.05.

## Results

### Manipulation checks

After performing the aerobic exercise program both with and without music, there were no significant differences in difficulty between the music group (*M* = 3.25, *SD* = 0.96) and the control group (*M* = 3.33, *SD* = 1.03), *t* (59) = -0.30, *p* = 0.77, *d* = 0.08; Levene’s test: *F* = 0.13, *p* = 0.72; 95% confidence interval: -0.59–0.44. In addition, no significant differences were found in comfortable perceived between the music group (*M* = 4.23, *SD* = 0.80) and the control group (*M* = 3.94, *SD* = 1.17), *z* = -0.87, *p* = 0.39, *r* = 0.11 (Mann–Whitney U Test with the data for comfortable).

However, the music group used a significantly lower amount of mental effort (*M* = 3.29, *SD* = 0.9) than the control group (*M* = 3.83, *SD* = 0.83), *t* (59) = -2.44, *p* = 0.02, *d* = 0.64; Levene’s test: *F* = 0.37, *p* = 0.55; 95% confidence interval: -0.99–0.10. The magnitude of the difference was consistent with Cohen’s (1992) definition of a medium-sized effect.

### Isometric grip task

As Table 3 shows, no significant differences were found in the change in duration from pre- to post-test between the two groups (ΔDuration), *t* (59) = -1.49, *p* = 0.14, *d* = 0.39; Levene’s test: *F* = 11.51, *p* = 0.001; 95% confidence interval: -3.74–0.57. However, the music group reported a statistically significant smaller increase in %MVC in post-test compared to the control group, *t* (59) = 2.27, *p* = 0.027, *d* = 0.59; Levene’s test: *F* = 2.79, *p* = 0.1; 95% confidence interval: 0.66–10.34. This was a medium-sized effect.

**Table 3:** Means and standard deviations for exercise planning and isometric grip task. *Mean* (*SD*).

	music(*n* = 31)	control(*n* = 30)
ΔRPE (RPE)	0.08 (0.30)	0.37 (0.42)
ΔDuration (s)	8.55 (3.16)	10.13 (4.99)
ΔEMG (%)	-22.97 (8.66)	-28.47 (10.19)

Note: (RPE) = ratings of perceived exertion (Borg, 1998), (s) = seconds

### Exercise planning

Levene’s test for ΔRPE data revealed significantly equal variances (*F* = 0.26, *p* = 0.61). The music group showed a significantly smaller decrease in the planned exercise from pre- to post-manipulation compared to the control group (ΔRPE), *t* (59) = -3.06, *p* = 0.003, *d* = 0.80; 95% confidence interval: -0.16–0.03 (Table 3). This was a large-sized effect [69].

## Discussion

This study is the first to explore the impact of accompaniment music on self-regulation depletion during aerobic exercise. The manipulation check showed that manipulation was effective in inducing different levels of self-regulation strength. The control group was seen as having more mental effort and self-regulation strength. After completing the aerobic exercise, the planned exertion (ΔRPE) was reduced and the EMG activation (ΔEMG) was increased; compared with the control group, the changes in the music group were smaller in both ΔRPE and ΔEMG. Self-regulation strength was quantified by isometric grip task and exercise planning tasks; previous studies show that after self-regulation depletion tasks, participants increased their EMG activation and reduced the exertion of their planned exercise [42, 43]. This shows that the consumption of self-regulation strength in aerobic tasks differed between the two groups: the music group was lower than the control group.

There was no statistically significant difference in ΔDuration between the groups. However, the iEMG activation of the control group increased more than that of the music group. Bray et al. [43] demonstrated that self-regulatory depletion has an effect on EMG activation. To maintain the same exercise performance (force generation), the depletion group needed a higher degree of iEMG compared to the control group. Accordingly, in the current study, it is thought that in order to maintain the same exercise performance (ΔDuration), the no-music group showed higher iEMG activation, which indicates that compared with the music group, the no-music group had depleted more self-regulation strength. Therefore, it is possible that music reduced the consumption of self-regulation strength in aerobic exercise. The positive effects of music on aerobic exercise were largely attributable to the effect of reduced the consumption of self-regulation strength.

Studies have shown that when they feel depleted, people expect to consume less self-regulation strength in their future plans [70, 71]. Our study found that the music group less decreased significantly in the exercise planning task (ΔRPE), compared to the control group. This shows that the music group consumed less self-regulation strength and decided to reduce the effort they were willing to devote to the planned exercise less than the control group. It is also indicated that aerobic exercise with music can affect an individual’s subsequent exercise attitude. This study is the first to show that music affects an individual’s exercise decision. Our findings extend the impact of music on individuals during aerobic exercise (e.g., [14]) and suggest that aerobic exercise accompanied by music may have an impact on subsequent exercise tasks and plans.

However, it is worth noting that there was no statistically significant difference in ΔDuration between groups. This may be because of the carryover effect caused by exercise fatigue. In other words, the ΔDuration differences between the groups were minimized by exercise fatigue, which contributes to the self-regulation depletion effect that cannot be detected.

This study showed that music during aerobic exercise can alleviate the consumption of self-regulation strength. However, similar self-regulation depletion has also been observed in HIIT and resistance exercise [7, 39, 40]. Other studies have shown that music has a positive impact on peoples’ affects in these two exercise modes [36, 37]. Thus, the outcomes of aerobic exercise may be extended to HIIT and resistance exercise. This needs to be explored further in future studies.

This study had some limitations. First, the sample consisted of individuals who were more active in physical activity. According to the Physical Activity Rating Scale-3, both groups of participants constituted individuals who engaged in a large amount of physical activity (> 43 indicates a large amount of exercise) [49]. Individuals with high levels of voluntary activity are more motivated to exercise. Muraven et al. [72] argued that motivation can diminish the depletion of self-regulation; thus, our study may overestimate the influence of music on self-regulated depletion in individuals who lack exersise motivation. Second, this study explored the impact of accompanying music on exercise intensity and duration after consuming self-regulation strength. The main components of exercise prescriptions are duration, intensity, frequency, and type. The lack of research on the other components of music has led to limitations in its application.

### Practical implications

As we can see, listening to music during aerobic exercise can be recommended for active individuals. The present findings demonstrate that music has the potential to reduce self-regulation depletion during exercise. Furthermore, the acute effects of self-regulation during exercise predicted exercise adherence over the following two months, and participants who reported smaller acute effects of depletion between pre- and post-tests were less likely to adhere to their future exercise plans [42]. Based on the findings of this study, music could reduce the acute effects of self-regulation depletion. These outcomes have been linked to increased adherence to exercise. Therefore, the application of music in aerobic exercise may be a practical strategy to help people get more out of their exercise, and can be used to encourage people to continue engaging in exercise.

## Conclusions

This study showed that aerobic exercise with music influences subsequent exercise states. Compared to the control group, the music group produced less neuromuscular (EMG) activity, which is indicative of less fatigue. In addition, the music condition increased the participants’ planned exercise exertion. Based on the strength model of self-regulation, the participants in the music group consumed less self-regulation strength during aerobic exercise.

## Supporting information

S1 Dataset (SAV)
